# Marine Polysaccharide Networks and Diatoms at the Nanometric Scale

**DOI:** 10.3390/ijms141020064

**Published:** 2013-10-09

**Authors:** Vesna Svetličić, Vera Žutić, Galja Pletikapić, Tea Mišić Radić

**Affiliations:** Division for Marine and Environmental Research, Ruđer Bošković Institute, Bijenička 54, 10000 Zagreb, Croatia; E-Mails: zutic@irb.hr (V.Ž.); gpletik@irb.hr (G.P.); tmisic@irb.hr (T.M.R.)

**Keywords:** atomic force microscopy, marine diatoms, extracellular polymeric substance, extracellular polysaccharides, marine gel network, self assembly, self-organization, *Cylindrotheca closterium*, *Bacteriastrum jadranum*, northern Adriatic Sea

## Abstract

Despite many advances in research on photosynthetic carbon fixation in marine diatoms, the biophysical and biochemical mechanisms of extracellular polysaccharide production remain significant challenges to be resolved at the molecular scale in order to proceed toward an understanding of their functions at the cellular level, as well as their interactions and fate in the ocean. This review covers studies of diatom extracellular polysaccharides using atomic force microscopy (AFM) imaging and the quantification of physical forces. Following a brief summary of the basic principle of the AFM experiment and the first AFM studies of diatom extracellular polymeric substance (EPS), we focus on the detection of supramolecular structures in polysaccharide systems produced by marine diatoms. Extracellular polysaccharide fibrils, attached to the diatom cell wall or released into the surrounding seawater, form distinct supramolecular assemblies best described as gel networks. AFM makes characterization of the diatom polysaccharide networks at the micro and nanometric scales and a clear distinction between the self-assembly and self-organization of these complex systems in marine environments possible.

## 1. Introduction

Diatoms, important marine photoautotrophic protists that account for up to 25% of the primary production on Earth [1], produce large quantities of extracellular polymeric substances (EPS), consisting predominantly of polysaccharides [2]. Diatom extracellular polymers participate in various processes, both at the cellular level and in the environment. At the cellular level, extracellular polymers have several important functions, of which some of the most frequently recognized are sessile adhesion, gliding, protection against drying, stabilization of habitats through prevention of sediment erosion, and the formation of biofilms and colonies [2,3]. The total amount of exopolysaccharides produced by diatoms is far in excess of that required for movement or adhesion [3–5] and is referred to as the photosynthetic overflow. In the marine environment, extracellular polysaccharide production by diatoms is a significant route by which photosynthetically produced organic carbon enters the trophic web and may influence the physical environment in the sea. Specifically, species of diatoms in the northern Adriatic Sea can produce large amounts, up to 50 g/m^3^ of extracellular polysaccharides in a month [6], resulting in the episodic formation of a macroscopic gel phase [7,8].

Marine polysaccharide gels represent a form of molecular organization in which biopolymer molecules form solvated three-dimensional networks imbedded in seawater. Intense metabolic activities of microorganisms within gel aggregates (e.g., [9]) result in the formation of sharp microbiogeochemical gradients. The diffusion-slowing properties of the gel phase aid in maintaining these gradients over a very small (μm) spatial scale. The continued presence of sharp microbiogeochemical gradients results in the formation of microbial consortia, structured arrangements of microorganisms exhibiting different but highly specific physiological activities over small spatial scales.

Unlike chemical gels that are formed by chemical reaction using a cross-linking agent, it is characteristic of anionic polysaccharide macromolecules to form gels by physical bonds through the intermolecular forces among polymer chains [10,11]. Among the new methodologies (experimental and theoretical) developed for and applied to polysaccharide conformation and dynamics, solution properties, chain aggregation and gelation [12] using atomic force microscopy (AFM) have yielded the most striking results [13].

AFM has been extensively used in nanoscale studies of EPS intimately associated with frustules as coating and adhesive structures (strands, tethers, pads and stalks), as recently reviewed by Higgins and Wetherbee, 2012 [14]. Here, we intend to review recent AFM studies of diatom EPS focusing on the detection of the supramolecular structures of polysaccharide fibrils produced by marine diatoms, either attached to the diatom cell wall or released into the surrounding seawater.

## 2. Basic Principles of AFM and Its Application to Polysaccharides

AFM connects the nanometer- and micrometer-length scales, utilizing a sharp probe that senses interatomic forces acting between the surface of a sample and the atoms at the apex of the tip. The physical basis behind AFM and its ability to “feel” the surface make AFM a versatile tool in biophysics, allowing high resolution imaging, nanomechanical characterization and measurements of inter- and intramolecular forces in living and non-living structures [15–19]. Thanks to the simple principle on which it is based, the AFM is a surprisingly small and compact instrument. Its use includes an electronic control unit, computer and usually two monitors for the simultaneous checking of the image and imaging parameters. The probe, which scans the sample surface, consists of a cantilever and a tip located at its free end. The deflection of the cantilever is measured by an optical detection system. Registered values of cantilever deflection are electronically converted into a pseudo 3D image of a sample. As a result AFM produces real 3D images of a sample with a vertical resolution of 0.1 nm and lateral resolution of 1 nm. The measured forces range from 10^−6^ N to 10^−11^ N. In AFM force spectroscopy, a single molecule or fiber is stretched between the AFM flexible cantilever tip and a flat substrate. A polysaccharide molecule, protein or other biopolymer is either adsorbed to the substrate or linked to it through the formation of covalent bonds. When the tip and substrate are brought together and then withdrawn, one or more molecules can attach to the tip. The deflection of the cantilever measures the force on the polymer with an accuracy of ~5 pN, while the piezoelectric positioner records the changes in the end-to-end length of the molecule with an accuracy of 0.1 nm. AFM force spectroscopy is a widely used method in polymer biophysics, allowing the measurement of the mechanical properties of single molecules with the possibility of quantifying the forces involved directly in both intra- and inter-molecular polymer interactions [18,20–23]. It has also been adopted in advancing diatom research into the nanotechnology [14].

In the AFM imaging mode that makes visualization at the molecular scale of polysaccharide samples possible [24–32], the molecules are usually spread on freshly cleaved mica (a hydrophilic aluminosilicate mineral). The imaging of hydrated samples is preferably conducted in air to inhibit the unfavorable motion of polysaccharides in liquid medium. Protocols for marine sample AFM imaging have been recently developed for single diatom cells and released polymers, isolated polysaccharides from diatom cultures and polymer networks of the marine gel phase [33–39].

## 3. AFM Studies of Diatom EPS

Higgins and coworkers [40] were the first to use AFM for the characterization of diatom extracellular polymers. Intra- and intermolecular forces were measured between polymers on the cell wall and between those extruded from the diatom raphe on diatom species *Craspedostauros australis* E.J. Cox and *Pinnularia viridis* (Nitzsch) Ehrenberg [41,42]. The resulting force curves obtained from the pores of cell walls were attributed to the soft and compressible material. However, due to their complexity, it was difficult to assign specific interactions between biopolymers to patterns on the force curves. AFM force spectroscopy was also applied to study extracellular adhesive pads released by the diatom *Toxarium undulatum* Bailey [43–45]. These pads are very sticky and cells use them to form colonies or attach themselves to the surface. The resulting force profiles with numerous sawtooth patterns (e.g., [46]) were attributed to the extensible modular proteins that are associated into the nanofibers. In a differently designed experiment, Arce and coworkers [47] compared the adhesion of whole diatom *Navicula* spp. cells on different surfaces. In that study, individual cells were glued to tipless cantilevers. With such “diatom probes,” standard force curves were recorded on surfaces such as Intersleek (a hydrophobic agent inhibiting biofilm formation) and mica. The resulting force curves showed comparable adhesion forces, and it was concluded that the extracellular polymeric material on the surface of *Navicula* spp. has both hydrophobic and hydrophilic properties. Direct imaging of EPS molecules was, however, hampered by the weak interaction of strands and tethers with the substrates used in the liquid [41]. These difficulties were overcome by using mica as a substrate and by imaging in air under ambient conditions (experimental details in [35]).

### 3.1. EPS of *Cylindrotheca* Species

The ubiquitous marine diatoms of the *Cylindrotheca* spp. were used in AFM studies of exopolysaccharide production [35–38]. The *Cylindrotheca closterium* (*Ceratonea Closterium* Ehrenberg) strain CCNA1 [35] was isolated from northern Adriatic seawater, while the CCMP1544 and *Cylindrotheca fusiformis* Reimann & J.C. Lewin, CCMP343 strains were obtained from the Bigelow Laboratory for Ocean Sciences.

The molecular organization of the EPS biopolymers released by *C. closterium* was approached at different levels: (i) EPS released by a single cell; (ii) EPS released in the culture medium and (iii) as biofilms grown on mica slides inserted in the culture [35]. The release of extracellular polymers by single cells was investigated by AFM in exponential and stationary growth phases. While the release of extracellular polymers in the exponential phase of growth was negligible, in the stationary phase extracellular polymers were visualized on more than 25% of the cells. The AFM data are in line with literature data on increased production of extracellular polymers in the stationary growth phase [48–52]. Parallel experiments with Alcian Blue staining and light microscopy performed in the cell culture have shown that the fibrils extending from the cell rostrum were mainly polysaccharides that existed in the liquid phase before the cell deposition to the mica surface [53].

**Figure 1:**
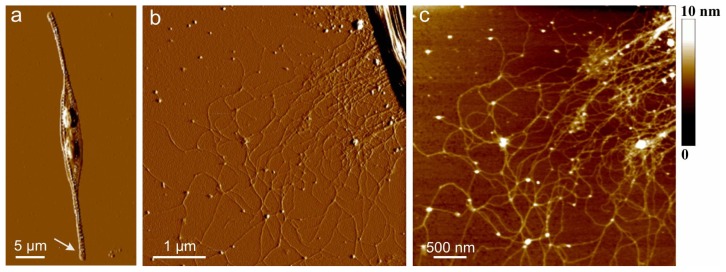
Extracellular polymers released by *C. closterium* (CCNA1) obtained by AFM imaging in contact mode after deposition on mica surface. (**a**) AFM image of the whole cell presented as deflection data. The arrow indicates the position of the polymer excretion site; (**b**) The released polymers still attached to the apex of the cell rostrum, deflection data, scan size 5 μm × 5 μm; (**c**) Released polymers presented as height data, scan size 4 μm × 4 μm and vertical scale shown as the color bar (reproduced from [33]).

AFM images of extracellular polymers released by *C. closterium* are shown in Figure 1. The image of the whole cell (Figure 1a) presents the general features of the *C. closterium* cell with two chloroplasts and its drawn-out flexible rostra. The arrow indicates the position of polymer release shown in b and c of Figure 1. Bundles of polymer fibrils appear at the position close to the site of excretion. Their heights were 5–7 nm. These bundles unfold into a fibrillar network with gradually decreasing fibril heights reaching a distance of up to 10 μm from the cell wall. At the distance of 1 μm, a dense network is observed with fibril heights of 2–3 nm. At larger distances, *C. closterium* EPS appeared as a relaxed network of fibrils with incorporated spherical nanoparticles/globules. The fibrils appeared flexible, usually with a curved shape, some of them even forming loops. The globules were found to interconnect two or more fibrils but were also imbedded along a single fibril. The fibril heights were in the range of 0.6 to 1.2 nm, while the average height of the incorporated globules was 5 nm. It was hypothesized that the globules are positively charged proteins whose function is the intracellular packing of negatively charged polysaccharide fibrils [35].

### 3.2. Biofilm of *Cylindrotheca Closterium*

Although several AFM studies exist that explored the nature of diatom biofilms and adhesives [40,45,47,54], visualization of a biofilm has only been achieved by AFM imaging in air [35]. The mica slides were withdrawn from the flasks when the cultures of diatom *C. closterium* entered the stationary growth phase (after 18 days), washed with ultrapure water and imaged in air after drying.

**Figure 2:**
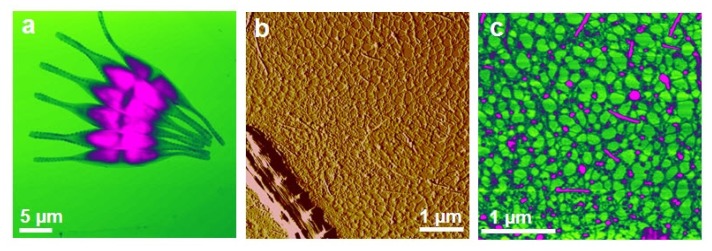
AFM image of biofilm grown in a CCNA1 culture formed on a mica slide. (**a**) Association of cells in the biofilm (height data, vertical scale 1.5 μm); (**b**) Cell rostrum surrounded by the biofilm network (deflection data); (**c**) The biofilm network (height data, vertical scale 10 nm). Images were acquired in contact mode (reproduced from [35]).

**Figure 3:**
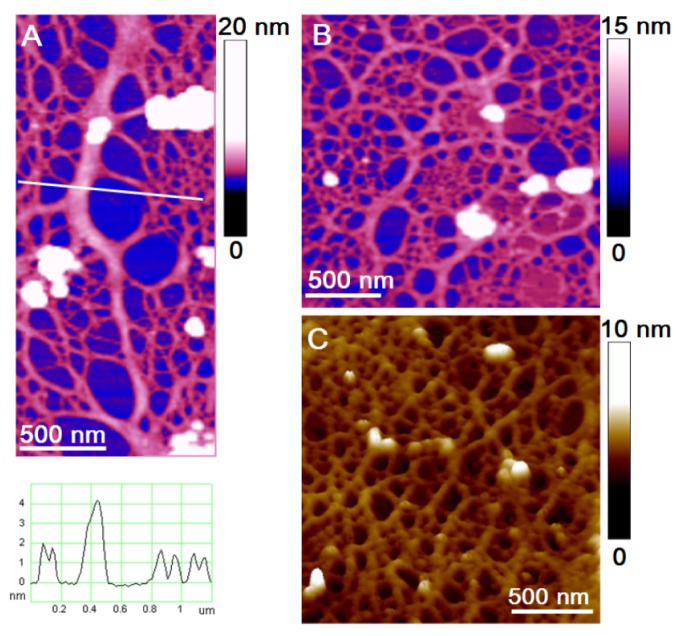
Structural details of the cell jacket network obtained by AFM. (**A**) branching fibril with height analysis along the indicated line; (**B**) 2D network of interconnect patches; (**C**) Spatial arrangement of interconnected patches in the 3D collapsed network (3D view). Images were acquired in tapping mode (reproduced from [39]).

**Figure 4:**
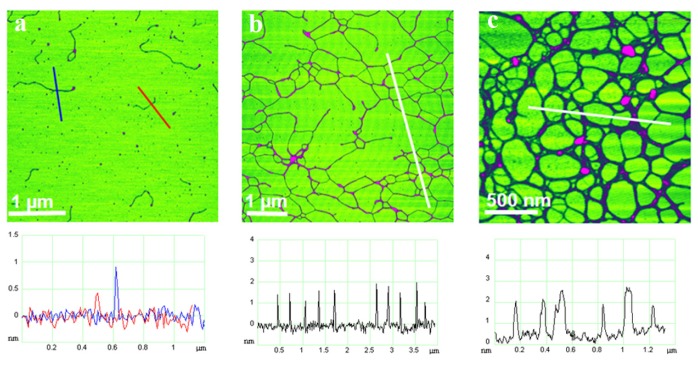
AFM images of polysaccharides isolated from the CCNA1 culture and dissolved in ultrapure water: (**a**) Single fibrils (concentration 5 mg/L) vertical scale 2.5 nm; (**b**,**c**) Fibril networks (concentration 10 mg/L), vertical scales: 5 nm (**b**) and 10 nm (**c**). The height analyses are shown along the indicated lines. The images were acquired in tapping mode and are presented as height data (reproduced from [35]).

**Figure 5:**
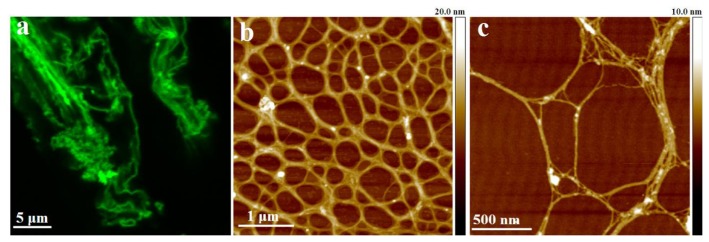
Marine gel fibrils at micro and nanometric scales: (**a**) A polysaccharide network of marine gel aggregate imaged in seawater by confocal microscopy after FITC-Concavalin A staining; (**b**,**c**) High resolution AFM images of marine gel fibrils with different degrees of cross-linking. AFM images were acquired in tapping mode in air using mica as a substrate (reproduced from [53]).

**Figure 6:**
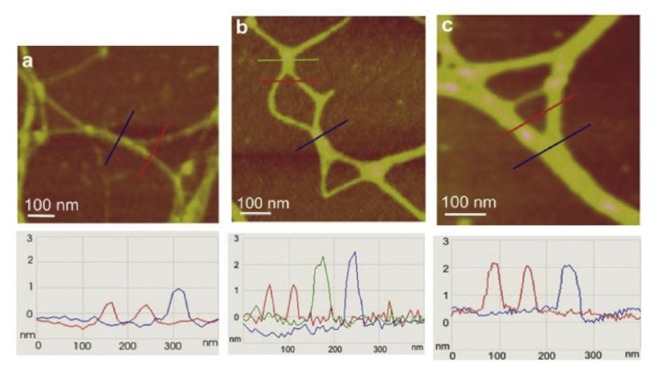
Mode of fibril associations into junction zones obtained by high resolution AFM imaging: (**a**) Two 0.7 nm high fibrils forming a 260 nm long junction zone; (**b**) Two 1.3 nm high fibrils forming 70 and 120 nm long junction zones; (**c**) Side-by-side association of two 1.6 nm high fibrils. The images were acquired in contact mode (adapted from [34]).

The observed biofilm appeared as a continuous fibrillar network between predominantly individual cells (Figure 2). Some cells were associated (Figure 2a) and 2–6 cells were captured on a 35 μm × 35 μm scanned biofilm surface. At a higher resolution, the biofilm was visualized as a dense fibrillar network with pore sizes ranging from 50 nm to 300 nm (Figure 2b,c). A gradual increase in pore sizes was detected going from the cell wall to the distance of 10 μm (Figure 2b). The globules (3–12 nm high) found exclusively on the fibrillar network appear as silica nanoparticles nucleated from the culture medium. Shchipunov and coworkers applied AFM to demonstrate that gel-forming polysaccharides (e.g., carrageenans) promote silica precipitation, serving as a template [55,56]. Comparison of the fibril and globule height analyses in the EPS of the two *C. closterium* strains and the biofilm grown in a *C. closterium* (CCNA1) culture is given in Table 1.

### 3.3. Polysaccharide Network in Diatom Colony Formation

Diatoms have evolved a variety of colonial life forms in which cells are connected by organic threads, mucilage pads or silicate structures. In general, these connecting structures are clearly visible by light or electron microscopy. The colonial planktonic diatom *Bacteriastrum jadranum* Godrijan, Marić & Phannkuchen [57] was found to be unusual, since the chain formation does not involve fusion of the setae of adjacent cells [39], unlike all other known colonial *Bacteriastrum* species [58]. Moreover, no thread or any other organic or inorganic substance clearly visible by light or electron microscopy connects the cells.

AFM imaging [39] provided the first evidence that *B. jadranum* cells in colonies are enclosed in a fibrillar polysaccharide network, termed a cell jacket. At nanoscale resolution, the cell jacket appeared as a cross-linked fibrillar network organized into recognizable patterns (Figure 3). Circular high-density domains (patches, Figure 3B), were surrounded and interconnected by thicker fibrils in a continuous network and appear as the basic structural motive. Pores inside a patch were of the same hexagonal shape, 8–100 nm in size. Their size was continuously smaller from the patch edge toward the center. The branching fibrils (Figure 3A) can be considered as the backbone of the network, critical for assuring its integrity. The pore-forming fibrils within the patches were only 0.6–1.6 nm high, the surrounding fibrils connecting the patches were 2.0–2.8 nm high, while the branching fibrils were considerably wider but not higher than 4.0 nm. Quantitative analysis of the cell jacket network is given in Table 2.

It was concluded that the *Bacteriastrum* polysaccharide jacket represents an essential part of the cell, as the conjunction of the polymer network with the frustule appears to be extremely tight and such specific and unique patterns were not found in the polysaccharide networks of marine gel imaged by AFM.

## 4. AFM of Marine Gel

The broad polydispersity of marine gels ranges from microscopic to macroscopic dimensions [59]. Santschi and coworkers [60] were the first to use AFM to image individual fibrillar polysaccharides in marine macromolecular organic matter. The massive appearance of gelatinous macroaggregates known as mucilage events [7,8,61] offered a possibility for systematic studies of the marine gel phase using AFM [53,62]. The macromolecular characterization of the isolated polysaccharide fraction using physico-chemical techniques [63] showed that they are polydisperse high-molecular weight heteropolysaccharides, in which at least some of the hydroxyl groups of sugar residues are substituted by ester sulfate groups and to a minor extent by carboxylic groups-uronic acid.

These features, conferring a marked polyelectrolytic behavior of the polysaccharides in salt solutions, were also found for the polysaccharide fraction from *C. closterium* EPS [36]. The fact that the EPS isolated polysaccharide fraction has the capacity to self-assemble into a gel network (Figure 4) is an important finding, with implications on the mechanism of gel phase formation in marine systems [36].

### 4.1. Imaging a Polysaccharide Network of Marine Gel

Development of the protocol for the AFM imaging of the marine gel phase was a critical step. The sampling procedure and specimen preparation protocol for AFM imaging is described in detail by Mišić, Radić *et al.* [34]. The main organizational features of the polymer gel network were preserved during the transfer of marine gel from seawater to a mica substrate in air. The appearance of a marine gel network at micro and nanometric scales is shown in Figure 5. The gel network imaged by AFM is to a certain extent distorted from the 3D structure in the aqueous phase due to attachment and stretching on the mica surface. Nevertheless, such constraints make studies of fibril associations at the molecular level that would not be accessible by other techniques possible. AFM imaging of marine gel provided insight into the molecular organization of the gel network and associations between polysaccharide fibrils forming the network. Among the complex network structures, associations of fibrils forming junction zones were identified. Modes of fibril associations into junction zones are exemplified in Figure 6.

**Figure 7:**
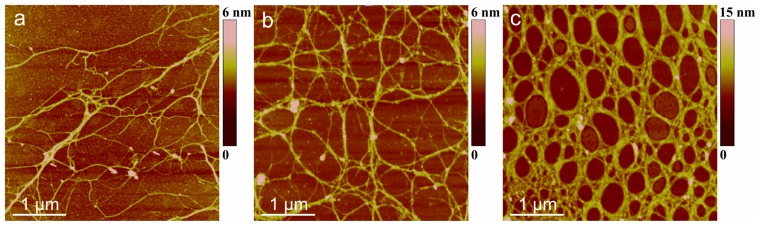
Evolution of polymer networks in the macroscopic gel phase from the early stage of gel phase formation (**a**,**b**) to the condensed gel network of an older macroaggregate (**c**). The AFM images were acquired in contact mode and presented as height data, scan size 4 μm × 4 μm (reproduced from [36]).

The evolution of polysaccharide fibrils into marine gel during the mucilage event in the northern Adriatic Sea was captured by AFM [36]. The samples were prepared from macroaggregates collected after different residence times in the water column, from the early stage of gel phase formation to the condensed (mature) gel network of an older macroaggregate. The long polymer strands with small patches of initial fibril associations (Figure 7a) coexisted with the continuous gel network shown in Figure 7b. With a prolonged residence time (one month), a more condensed network was formed, as presented in Figure 7c.

### 4.2. Force Spectroscopy of a Marine Gel Network

The knowledge of the mechanical strength of individual molecular assemblies within a marine gel network contributes to the understanding of the gel phase formation and its persistence in the marine environment. Due to the inherent complexity and heterogeneity of the marine gel phase, it is difficult to isolate the physical forces in the biopolymer network assemblies. However, based on AFM imaging and differential scanning calorimetry, the marine gel was characterized as a thermoreversible physical gel and the dominant mode of gelation was proposed to be the crosslinking of polysaccharide fibrils by hydrogen bonding, which results in helical structures and their associations [34].

**Figure 8:**
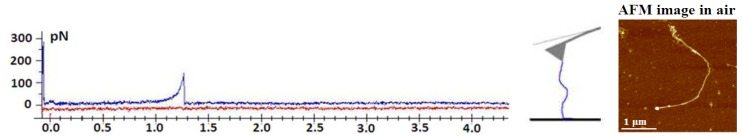
Force curves of a single polysaccharide fibril from a disentangled gel network (AFM image shown as the insert) acquired in filtered seawater: approach curve in red and extension curve in blue (adapted from [63]).

**Figure 9:**
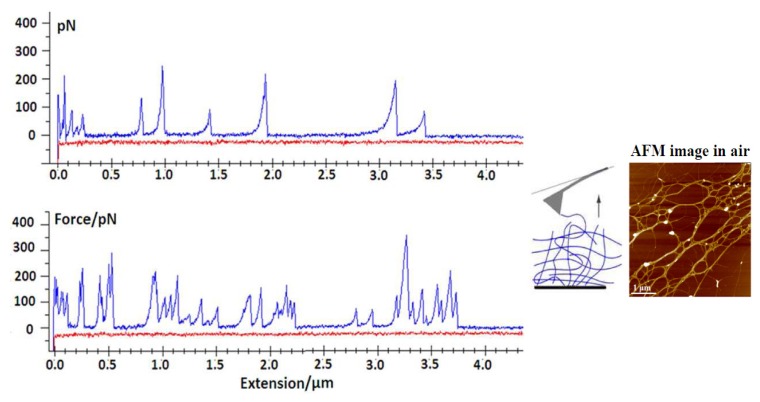
Force curves of polysaccharide fibrils in a marine gel network (AFM image shown as the insert) acquired in filtered seawater: approach curve in red and extension curve in blue (adapted from [63]).

Force spectroscopy and high resolution AFM imaging were applied to quantify the intramolecular, interdomain and intermolecular forces within the marine gel network [63,64]. The ability to control the degree of gel network entanglements by dilution and stirring was used to probe marine polysaccharides at different levels of association. The typical events that lead to specific patterns upon stretching include the entropic behavior of individual fibrils (Figure 8) and more complex events, such as the unfolding of polysaccharide entanglements (Figure 9).

Marine gels are highly extensible, as they may be stretched with very little force to distances of up to several micrometers. Fairly large forces are required to unzip the fibrils, suggesting that lateral stability may be important in maintaining the structural integrity of the marine gel.

## 5. Conclusions

AFM made characterization of diatom polysaccharide networks at the nanometric scale and a clear distinction between the self-assembly and self-organization of these complex systems possible. Marine polysaccharides produced by diatoms are shown to form distinct supramolecular assemblies that are best described as gel networks. These photosynthetically produced macromolecules may stay attached to the cell wall or be released into the environment.

This review encompasses three specific examples of polysaccharide polymer networks attached to the diatom cell wall: (i) *Cylindrotheca closterium* polymers at the moment of their release; (ii) a *C. closterium* biofilm grown on an atomically smooth substrate and (iii) a 3D polysaccharide network enclosing a cell colony of planktonic diatom *Bacteriastrum jadranum*.

Extracellular polymers produced by *C. closterium* exhibit a certain level of self-organization, while the highest level of self-organization was found in a 3D polysaccharide network produced by *B. jadranum*. The polysaccharide fibrillar network of *B. jadranum* represents an essential part of the cell and not a random extracellular polysaccharide structure. With a more generalized use of AFM, it would probably be discovered that there are many similar types of coverings among planktonic diatoms, as well as diatoms adapted to other habitats (e.g., benthos, tychoplankton *etc.*). This may be additionally extended to a wider range of microalgae and protists.

In contrast to the networks attached to the diatom cells, free-floating marine gel aggregates are formed by a self-assembly of diatom-released polymers. Structural details of the gel network visualized down to the molecular level revealed monomolecular, helical and superhelical associations.

The physical forces in marine gel network assemblies have been quantified using force spectroscopy together with high resolution AFM imaging. The marine gels appeared highly extensible, as they could be stretched with very little force to distances of up to several micrometers. Such self-assembled networks with randomly distributed microscopic features and high extensibility are capable of responding to environmental conditions, such as change in salinity, temperature, pH and shear stress, maintaining favorable physiological conditions for microbial communities.

## Figures and Tables

**Table 1:** Height ranges of the fibrils and globules in the EPS and biofilm of *C. closterium* strains CCNA1 and CCMP1544 (data from [35]).

Cell culture	Fibril height/nm			Globule height/nm		
	EPS bound to cell	EPS in bulk culture	Biofilm	EPS bound to cell	EPS in bulk culture	Biofilm
CCNA1	0.4–1.9	0.4–2.2	1.7–4.0	3–12	2–12	3–12
CCMP1544	0.6–1.6	0.7–2.6	–	3–9	2–13	–

**Table 2:** Cell jacket network: pores and pore-forming fibrils analyzed over a surface area of 4 × 4 μm^2^ (data from [39]).

Pores		Pore-forming fibrils

size/nm	number	height/nm
8–100	900	0.6–1.6
100–160	200	2.0–2.8
500–1000	10	2.5–4.0
